# European follow-up of incorrect biomarker results for colorectal cancer demonstrates the importance of quality improvement projects

**DOI:** 10.1007/s00428-019-02525-9

**Published:** 2019-02-05

**Authors:** Cleo Keppens, Kelly Dufraing, Han J. van Krieken, Albert G. Siebers, George Kafatos, Kimberly Lowe, Gaston Demonty, Elisabeth M. C. Dequeker

**Affiliations:** 1 Department of Public Health and Primary Care, Biomedical Quality Assurance Research Unit, University of Leuven, Kapucijnenvoer 35 block d, 1st floor, box 7001, 3000 Leuven, Belgium; 2 Department of Pathology, Radboud University Medical Center, Geert Grooteplein 10 (route 812), P.O.Box 9101, 6500 HB Nijmegen (824), The Netherlands; 3 Amgen Ltd, 1 Uxbridge Business Park, Sanderson Road, Uxbridge, UB8 1DH UK; 4 Amgen Inc, One Amgen Center Drive, MS 17-2-A, Thousand Oaks, CA 91320 USA; 5 Amgen Belgium S.A./N.V, Arianelaan 5, 1200 Brussels, Belgium

**Keywords:** Colorectal cancer, External quality assessment, Molecular pathology, Error analysis, Corrective actions, Biomarker analysis

## Abstract

Biomarker analysis for colorectal cancer has been shown to be reliable in Europe with 97% of samples tested by EQA participants to be correctly classified. This study focuses on errors during the annual EQA assessment. The aim was to explore the causes and actions related to the observed errors and to provide feedback and assess any improvement between 2016 and 2017. An electronic survey was sent to all laboratories with minimum one genotyping error or technical failure on ten tumor samples. A workshop was organized based on 2016 survey responses. Improvement of performance in 2017 was assessed for returning participants (*n* = 76), survey respondents (*n* = 13) and workshop participants (*n* = 4). Survey respondents and workshop participants improved in terms of (maximum) analysis score, successful participation, and genotyping errors compared to all returning participants. In 2016, mostly pre- and post-analytical errors (both 25%) were observed caused by unsuitability of the tumor tissue for molecular analysis. In 2017, most errors were due to analytical problems (50.0%) caused by methodological problems. The most common actions taken (*n* = 58) were protocol revisions (34.5%) and staff training (15.5%). In 24.1% of issues identified no action was performed. Corrective actions were linked to an improved performance, especially if performed by the pathologist. Although biomarker testing has improved over time, error occurrence at different phases stresses the need for quality improvement throughout the test process. Participation to quality improvement projects and a close collaboration with the pathologist can have a positive influence on performance.

**Electronic supplementary material:**

The online version of this article (10.1007/s00428-019-02525-9) contains supplementary material, which is available to authorized users.

## Introduction

Metastatic colorectal carcinoma (mCRC) is the third most commonly diagnosed malignancy and the fourth leading cause of cancer death worldwide [1]. Besides standard chemotherapy, mCRC patients are currently receiving personalized treatment by anti-epidermal growth factor receptor (EGFR) monoclonal antibodies, which has been shown to significantly increase the median survival time from 18.5 to 23.5 months of mCRC patients [2].

In 2008, mutations in exon 2 of the Kirsten rat sarcoma viral oncogene homolog (*KRAS*) gene were shown to be a negative predictor for anti-EGFR therapy benefit [2, 3]. In 2013, the same was demonstrated for mutations in *KRAS* exons 3 and 4, and for the less frequent mutations in neuroblastoma rat sarcoma (*NRAS*) viral oncogene homolog exon 2–4 [4, 5], resulting in an extension of the drug labels for cetuximab and panitumumab by the European Medicine Agency (EMA) [6, 7]. Consequently, molecular diagnostic laboratories were challenged to include these new test requirements in a correct and timely manner [4].

Since 2009, the European Society of Pathology (ESP) has been involved in the organization of a yearly colon external quality assessment (EQA) scheme to assess and improve *RAS* biomarker analysis in mCRC [8, 9]. Based on the updated requirements, the 2013 ESP colon EQA scheme was expanded by the assessment of full *RAS* testing (exon 2, 3, and 4 of both *KRAS* and *NRAS*) [10]. Since that same year, laboratories could also optionally test the *BRAF* (B-Raf proto-oncogene) gene, which has demonstrated prognostic value and is increasingly being analyzed in Europe [11].

Results from the 2013 scheme revealed that full *RAS* testing was only implemented by half of the laboratories (49.3%, *n* = 131 laboratories) and that there were numerous errors in testing the new gene segments [10]. In addition, EQA data confirmed that molecular diagnostic laboratories in Europe are using a large variation in methods for (a) the estimation of the neoplastic cell content [12], (b) for DNA extraction [13], and (c) for determining the *RAS* and *BRAF* status [13].

A 2016 study showed that the vast majority of samples (97%) tested by laboratories participating to an EQA scheme had been correctly classified. For about 2% of samples tested, an incorrect outcome was obtained that could potentially lead to a different anti-EGFR therapy advice [14]. Given the potential impact of predictive biomarker analyses on patient outcome, it is important to evaluate the exact causes of errors and to provide tailored feedback to diagnostic laboratories for quality improvement [15]. In turn, laboratories are encouraged to implement the necessary corrective and preventive actions (CAPA) as a required by the ISO15189 standard [16] or national equivalents, and the Clinical Laboratory Improvement Amendments of 1988 [17].

In clinical biology and forensics, error causes have been shown to occur mostly during the pre- (46–86%) and post-analytical (18–47%) phases of the total test process (TTP) compared to the analytical phase (7–13%) [18, 19], although the lack of standardization in taxonomy accounts for some of the variation seen in these error rates [20].

Although EQA schemes reflect the performance of diagnostic laboratories, more detailed information is required on the error causes and distribution throughout the TTP for molecular cancer diagnostics, as well as the actions undertaken by laboratories to improve quality in the long-term [21].

Therefore, the objectives of this study were (a) to evaluate the causes, distribution, and follow-up of laboratory errors from laboratories participating to the ESP colon EQA scheme; (b) to provide feedback to laboratories as to how practice can be improved, and (c) to assess potential improvement between 2016 and 2017 EQA schemes.

## Material and methods

The 2016 and 2017 ESP colon EQA schemes were organized according to the ISO 17043 standard for proficiency testing [22] and the guideline on the requirements of external quality assessment programs in molecular pathology [23]. Participation to EQA was free of choice and open to all laboratories worldwide. Details on validation, results submission, and feedback provided to the laboratories have been previously described ([13], Supplemental Table 1).

At the end of both EQA schemes, all laboratories with at least one major genotyping error, a score “i,” or technical failure (in which no result could be obtained for a case) in one of the ten provided formalin-fixed paraffin-embedded cases were invited by e-mail to complete an electronic survey with both laboratory-specific (general) questions and case-specific questions for each observed error. A list of definitions was included to clarify all questionnaire terms (Supplemental Table 2). Data was collected for 1 month, laboratories received a first reminder after 14 days and a second the day before the deadline.

All participants to the 2016 EQA scheme were invited to attend a 1.5-day long optional workshop, organized in December 2016 at the Radboud University Medical Center, The Netherlands. Topics were based on the 2016 survey output and included issues occurring in the pre-, post-, and analytical phase as cited by the survey respondents. A separate microscopy session focusing on the estimation of neoplastic cell content was held outside this project (Dufraing et al., submitted for publication).

Improvement of *RAS* testing was evaluated between both ESP colon EQA scheme years on three levels: (a) laboratories who participated in both schemes, (b) 2016 survey respondents, and (c) participants to the 2016 workshop. For these three categories, the average genotyping score, percentage of participants with the maximum score of 20, the percentage of successful participants, and re-occurrence of genotyping errors and/or technical failures were assessed.

Response bias was assessed by investigating the difference in laboratory characteristics between survey respondents and non-responders. Missing data were reported in the tables accordingly and not included in the statistical analysis. The reported accreditation statuses and laboratory settings were validated on the websites of the relevant national accreditation bodies and the laboratories’ website, respectively. Comparison of categorical variables was performed using chi-squared (*Χ*^2^) tests or a Fisher’s exact (FE) test if one of the row or column cells counted below five. For the difference in the sample amounts tested and people involved in the laboratory between responders and non-respondents, categories were treated as ordinal data following a Mann-Whitney *U* (MWU) test. For a combination of categorical and continuous variables (e.g., improvement of the average genotyping score between more than two groups) a one-way ANOVA with Tukey’s HSD was performed. Bonferroni corrections were applied when necessary. The significance level was set at *α* = 0.05. All statistical analyses were performed using SPSS Statistics Subscription version 1.0.0.903 (IBM, Armonk, NY, USA). Graphs were created using Microsoft Excel Professional Plus 2013.

## Results

### ESP Colon EQA scheme results

In the 2016 and 2017 colon EQA schemes, 123 laboratories from 27 countries and 105 from 29 countries participated, respectively. Seventy-six laboratories participated in both EQA schemes. They displayed a significant correlation between the average genotyping scores in 2016 and 2017 (Spearman *R* = 0.29, *p* = 0.011). Participants with a genotyping error or the maximum score in 2016 were more likely to obtain a genotyping error (*p* = 0.032) and reach the maximum score (*p* = 0.022) in 2017.

In 2017, the average genotyping score was 82.5% and the successful participation according to pre-defined scoring criteria [23] was 57.1% (60/105). In addition, 45.7% (48/105) laboratories obtained the maximum score of 20/20. In 2017, the number of participants making a genotyping error was 41.9% (44/105), whereas the number of participants with a technical failure was 3 participants (2.9%).

The total number of genotyping errors and technical failures on sample level is shown in Table 1.

**Table 1:** Overview of samples containing technical failures and genotyping errors in the ESP Colon EQA schemes, and the number of them addressed in the survey responses

	2016				2017			
	# samples in EQA scheme	% samples in EQA scheme	# samples in EQA scheme with survey feedback	% samples in EQA scheme with survey feedback	# samples in EQA scheme	% samples in EQA scheme	# samples in EQA scheme with survey feedback	% samples in EQA scheme with survey feedback
With technical failures	12/1230	1.0	8/12	66.7	4/1050	0.4	1/4	25.0
With genotyping errors	56/1230	4.6	24/56	42.9	64/1050	6.1	25/64	39.1
Type of genotyping error								
False-positive	14	1.1	8	57.1	8	0.8	6	9.4
False-negative	29	2.4	10	34.5	17	1.6	6	9.4
Wrong mutation	7	0.6	3	42.9	19	1.8	5	7.8
Samples switched	4	0.3	2	50.0	5	0.5	5	7.8
Score i	2	0.2	1	50.0	15	1.4	3	4.7
Sample variant status								
*KRAS*	28	2.3	9	32.1	33	3.1	13	20.3
*NRAS*	9	0.7	3	33.3	5	0.5	2	3.1
*BRAF*	6	0.5	3	50.0	4	0.4	2	3.1
WT	10	0.8	8	80.0	5	0.5	3	4.7
Sample without neoplastic cells	3	0.2	1	33.3	17	1.6	5	7.8

For the genotyping errors, a subdivison is made based on the type of error and variant status. False-positive: reported a variant in a wild-type sample, or an additional incorrect variant besides the correctly present variant; false-negative: wild-type reported in a sample containing a variant; wrong mutation: incorrect variant found in a sample containing a variant, in the same gene or in a different gene; technical failures: no conclusive results were reported by the participant; score i: sample analyzed and outcome given in a case without neoplastic cells. Reference sequences used at the time of analysis: *KRAS*: NM_033360.3, NM_004985.4. *NRAS*: LRG_92 (NM_002524.3), *BRAF*: LRG_299 (NM_004333.4). Abbreviations: *BRAF*, B-Raf proto-oncogene; *KRAS*, Kirsten rat sarcoma viral oncogene homolog; *NRAS*, neuroblastoma rat sarcoma; WT, wild-type

The proportion of samples misclassified was 4.6% (56/1230) in 2016 and 6.1% (64/1050 samples) in 2017. The average genotyping score, the number of participants with a successful participation and the number of technical errors were lower in the 2017 compared to the 2016 EQA scheme. The number of genotyping errors were higher in 2017, but not related to (a) new participants, (b) laboratories who switched methods, or (c) used a specific method type.

### Characteristics of survey respondents

Based on the EQA results, 51 and 49 laboratories received the survey in 2016 and 2017, respectively. Twenty-two (43.1%) participants in 2016 and 18 (36.7%) in 2017 responded within 20 days. Data from one survey participant in 2016 was not taken into account for further analysis, as only one of the questions was answered. Response rates did not differ depending on the country, or whether they had completed the previous (2016) survey.

The laboratory characteristics during the 2016 and 2017 EQA schemes are shown in Table 2. For both schemes most respondents performed *RAS* and *BRAF* analysis in a routine clinical setting using commercial kits. The majority were not accredited for molecular analysis and were situated in a university or general hospital. For most of the respondents the analysis was performed under the department of pathology (61.9% and 88.9% in 2016 and 2017, respectively), and included on average between 1 and 10 people. Six (28.6%) laboratories that participated in the 2016 scheme responded that the analysis was performed by another laboratory (compared to 0.0% in 2017). Of these, 5 laboratories outsourced the evaluation of the neoplastic cells, and 1 the DNA extraction step. For all participants, the estimation of the percentage of neoplastic cells was performed by a pathologist.

**Table 2:** Overview of laboratory characteristics for non-survey respondents and survey respondents as obtained during the EQA scheme

	2016				2017			
	# responders (*n* = 21°)		# non-responders (*n* = 101)		# responders (*n* = 18)		# non-responders (*n* = 87)	
Number of countries	14		26		14		25	
Performs test in routine practice								
*KRAS*	20	95.2	96	95.05	17	94.4	82	94.3
*NRAS*	20	95.2	94	93.07	17	94.4	81	93.1
*BRAF*	18	85.7	89	88.12	14	77.8	81	93.1
Number of *KRAS* samples tested in last 12 months								
1–99	4	19.0	18	17.82	5	27.8	21	24.1
100–249	8	38.1	43	42.57	5	27.8	35	40.2
250–499	5	23.8	24	23.76	7	38.9	17	19.5
> 500	3	14.3	11	10.89	0	0.0	9	10.3
No clinical testing	1	4.8	5	4.95	1	5.6	5	5.7
Number of *NRAS* samples tested in last 12 months								
1–99	6	28.6	24	23.76	10	55.6	24	27.6
100–249	6	28.6	43	42.57	4	22.2	37	42.5
250–499	5	23.8	21	20.79	3	16.7	15	17.2
> 500	3	14.3	6	5.94	0	0	5	5.7
No clinical testing	1	4.8	7	6.93	1	5.6	6	6.9
Number of *BRAF* samples tested in last 12 months								
1–99	7	33.3	40	39.60	9	50.0	37	42.5
100–249	5	23.8	32	31.68	3	16.7	28	32.2
250–499	4	19.0	12	11.88	2	11.1	13	14.9
> 500	2	9.5	5	4.95	0	0.0	3	3.4
No clinical testing	3	14.3	12	11.88	4	22.2	6	6.9
People involved in the analysis								
1–10	16	76.2	91	90.10	16	88.9	78	89.7
11–20	4	19.0	7	6.93	1	5.6	7	8.0
> 20	1	4.8	3	2.97	1	5.6	2	2.3
Laboratory setting								
Anti-cancer center	3	14.3	8	7.9	3	16.7	10	11.5
Education and research hospital	0	0.0	1	1	0	0.0	2	2.3
General hospital	8	38.1	26	25.7	5	27.8	25	28.7
Industry	0	0.0	3	3	1	5.6	5	5.7
Private	5	23.8	20	19.8	4	22.2	15	17.2
Private hospital	0	0.0	4	4	0	0.0	2	2.3
University	0	0.0	6	5.9	2	11.1	4	4.6
University hospital	5	23.8	33	32.7	3	16.7	24	27.6
Accreditation status								
Accredited	10	47.6	40	39.6	3	16.7	34	39.1
Not accredited	11	52.4	61	60.4	15	83.3	53	60.9
Analysis performed under the department of pathology	*Χ^2^(1) = 12.3, *p* < 0.001							
Yes	13	61.9	92	91.09	16	88.9	71	81.6
No	8	38.1	9	8.91	2	11.1	16	18.4
Part of the analysis performed by another laboratory	*Χ^2^(1) = 3.9, *p* = 0.05							
Yes	6	28.6	12	11.88	0	0.0	12	13.8
No	15	71.4	89	88.12	18	100.0	75	86.2
Method *KRAS*								
Commercial kit	10	47.6	49	48.51	11	61.1	40	46.0
NGS	5	23.8	24	23.76	3	16.7	27	31.0
Non-commercial method	6	28.6	28	27.72	4	22.2	20	23.0
Method *NRAS*								
Commercial kit	9	42.9	46	45.54	11	61.1	37	42.5
NGS	5	23.8	24	23.76	3	16.7	27	31.0
Non-commercial method	7	33.3	31	30.69	4	22.2	23	26.4
Method *BRAF*								
Commercial kit	7	33.3	39	38.61	9	50.0	35	40.2
NGS	5	23.8	22	21.78	3	16.7	25	28.7
Non-commercial method	5	23.8	26	25.74	2	11.1	18	20.7
Not performed	4	19.0	14	13.86	4	22.2	9	10.3

No missing data was observed for a specific question unless specified otherwise in the table. °1 laboratory was not included as a survey respondent because all data was incomplete. *Significant difference. Abbreviations: *BRAF*: B-Raf proto-oncogene, *KRAS*: Kirsten rat sarcoma viral oncogene homolog, *NGS*: next-generation sequencing, *NRAS*: neuroblastoma rat sarcoma

Laboratory characteristics collected during the EQA scheme for those participating in the survey were compared to the non-respondents. In 2016, the survey participants were less likely to have performed the analysis under the pathology department compared to non-participants. Similarly, the 2016 respondents were more likely to have outsourced the analysis to another laboratory (Table 2).

### Error cause analysis

Responses to the case-specific questions resulted in a total of 35 (2016) and 24 (2017) issues that were further analyzed. The issues examined in this study comprised 42.9% and 39.1% of the total genotyping errors and 66.7% and 25.0% of the technical failures observed in the 2016 and 2017 EQA schemes, respectively. An overview of case-specific and laboratory-specific answers is given in Table 3.

**Table 3:** Overview of survey responses after the 2016 and 2017 ESP colon EQA scheme

Question	2016 survey respondents		2017 survey respondents	
	# observations	% observations	# observations	% observations
Case-specific questions				
Total number of errors analyzed	35	100.0	24	100.0
Phase in the total testing process				
Pre-analytical	12	34.3	6	25.0
Analytical	10	28.6	12	50.0
Post-analytical	13	37.1	6	25.0
Type of problem				
Clerical error	6	17.1	2	8.3
Interpretation error	5	14.3	3	12.5
Methodological problem	3	8.6	7	29.2
Personnel error	5	14.3	6	25.0
Problem with the tissue	10	28.6	2	8.3
Reagent problem	2	5.7	0	0.0
Technical problem	3	8.6	4	16.7
Missing data	1	2.9	0	0.0
Detection of the error*	FE, *p* < 0.05			
Before release of the EQA results	1	2.9	6	25.0
After release of the EQA results	25	71.4	17	70.8
Missing data	9	25.7	1	4.2
Corrective/preventive actions*	*Χ*^2^ (9) = 18.6, *p* < 0.05			
Contact manufacturer	2	5.7	5	20.8
None	6	17.1	8	33.3
Optimization/implementation of documents	1	2.9	0	0.0
Protocol revision	15	42.9	5	20.8
Protocol revision + subsequent staff training	0	0.0	2	8.3
Retesting of samples	1	2.9	0	0.0
Staff training	6	17.1	3	12.5
Unknown	3	8.6	0	0.0
Missing data	1	2.9	0	0.0
Change method	0	0.0	1	4.2
Person involved in follow-up°	FE, *p* < 0.05			
Lead laboratory technician*	12	34.3	1	4.2
Laboratory technician	9	25.7	5	20.8
Pathologist	10	28.6	5	20.8
Molecular biologist	17	48.6	14	58.3
Quality manager	2	5.7	3	12.5
Laboratory director*	4	11.4	9	37.5
Scientific employee	1	2.9	0	0.0
Medical geneticist	0	0.0	1	4.2
Missing data	5	14.3	0	0.0
Laboratory-specific questions				
Total number of laboratories responded	21	100.0	18	100.0
General change of method/protocol based on the EQA results				
Yes	12	57.1	4	22.2
No	9	42.9	10	55.6
Maybe	0	0.0	2	11.1
Missing data	0	0.0	2	11.1
Person involved in interpretation of the results°				
Lead laboratory technician	3	14.3	0	0.0
Laboratory technician	8	38.1	6	33.3
Pathologist	8	38.1	6	33.3
Molecular biologist	15	71.4	15	83.3
Molecular biology consultant	0	0.0	1	5.6
Laboratory director	2	9.5	2	11.1
Clinical biologist (MD)	1	4.8	0	0.0
Engineer	1	4.8	0	0.0
Medical geneticist	0	0.0	1	5.6
Training of the personnel involved in interpretation of the result°				
By school degree	4	19.0	2	11.1
External: attending workshops	3	14.3	2	11.1
External: training by manufacturer	4	19.0	0	0.0
Internal and external (not specified)	1	4.8	0	0.0
Internal only (not specified)	1	4.8	0	0.0
Internal: exchange with other lab/EQA	1	4.8	0	0.0
Internal: learning from colleagues with gradually more independence	6	28.6	5	27.8
Internal: participation to laboratory meetings	4	19.0	1	5.6
Internal: performing validations	4	19.0	3	16.7
None	3	14.3	0	0.0
Missing data	0	0.0	6	33.3
Person involved in reporting of the results°				
Lead laboratory technician	2	9.5	0	0.0
Laboratory technician	2	9.5	4	22.2
Pathologist	10	47.6	6	33.3
Molecular biologist	11	52.4	12	66.7
Quality manager	1	4.8	0	0.0
Laboratory director	3	14.3	3	16.7
Clinical biologist (MD)	1	4.8	0	0.0
Medical geneticist	0	0.0	1	5.6
Administrative staff	0	0.0	1	5.6
Request for retesting the sample*	*Χ*^2^ (3) = 22.5, *p* < 0.001			
No	1	4.8	4	22.2
Yes, always	6	28.6	4	22.2
Yes for routine practice but not in EQA	1	4.8	10	55.6
Missing data	13	61.9	0	0.0

No missing data was observed for a specific question unless specified in the table. °Multiple options could be selected, which is why percentages add up to more than 100.0%. *Statistical difference

The majority of the 2016 errors (37.1%) occurred in the post-analytical phase of the testing process, compared to analytical problems in 2017 (50.0%). “Tissue problems” caused by the insuitability of the tumor tissue (e.g., insufficient amount of neoplastic cells or degradation of the DNA), and methodological problems were the most frequent detailed causes. In 2017, problems were more frequently detected before release of the final EQA results, and more often no CAPA was undertaken. Looking at all test phases, only laboratories with an error in the pre-analytical phase were less likely to obtain a maximum score in 2017 (*p* = 0.031). There was no difference in the number of errors in subsequent test phases and their specific causes compared to genotyping errors, technical failures, successful participations, or maximum scores obtained in 2017.

In both years, occurrence throughout the TTP differed significantly for different analysis methods (*KRAS p* = 0.019, *NRAS p* = 0.044, *BRAF p* = 0.006). Analytical and post-analytical errors occurred more for commercial kits, compared to pre-analytical errors for non-commercial users. However, specific error causes and the CAPAs undertaken were not linked to a certain methodology.

### Corrective/preventive actions

On average between 1 and 2 persons were included in carrying out the respective CAPA although errors in 2017 required more often involvement of the laboratory director compared to the (lead) technician in 2016 [Table 3]. There was no link between the phase or cause of the problem and the number or occupation of persons involved. However, only errors followed-up by the pathologist, were less likely to result in a genotyping error in 2017 and were more likely to result in a successful participation (both *p* = 0.012).

**Fig. 1:**
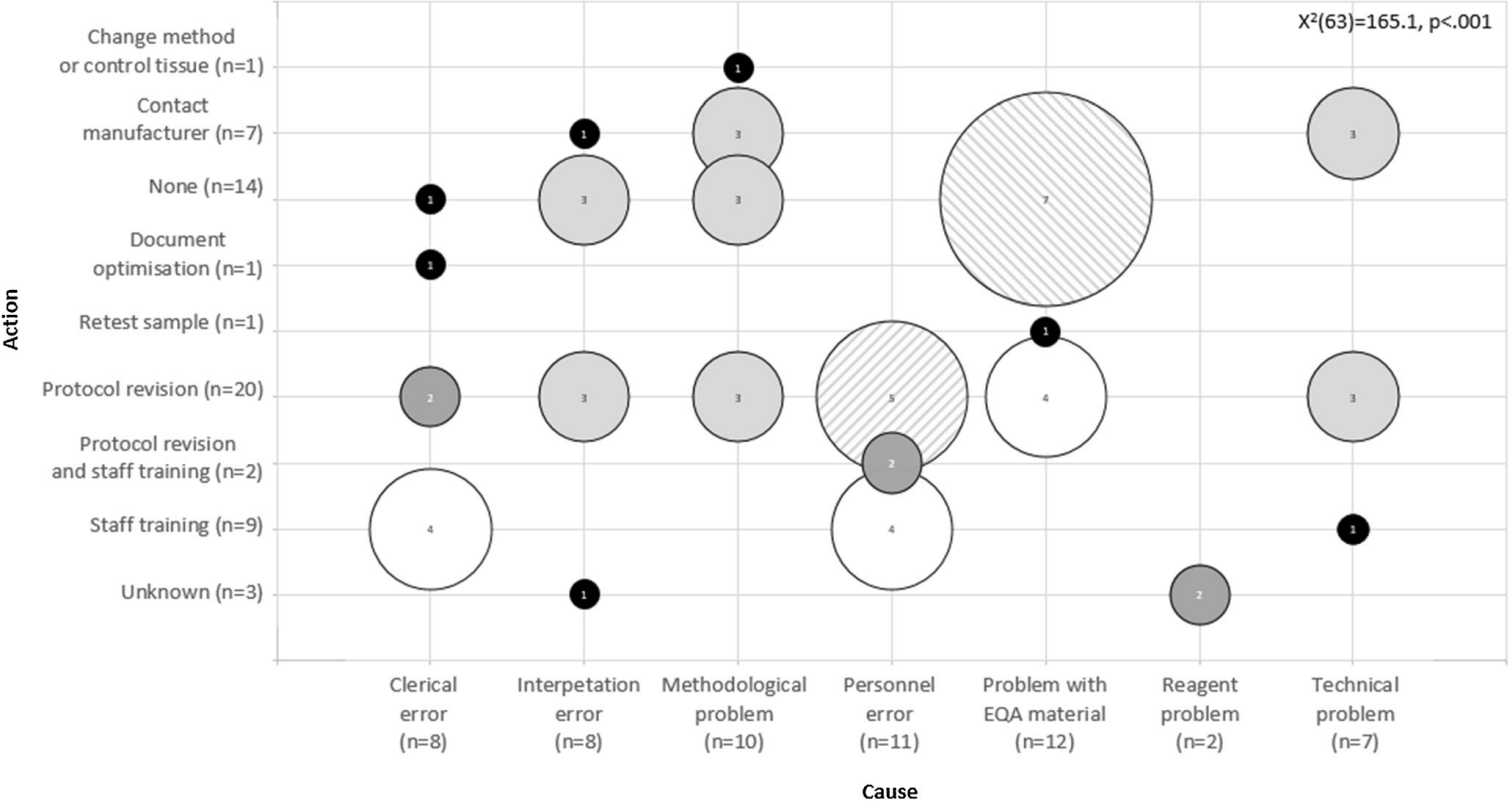
Overview of performed actions according to error causes reported by survey respondents in 2016 and 2017. The size of the bubbles represents the number of combinations between error causes and CAPAs

The most performed CAPAs included protocol revisions (*n* = 20) and staff trainings (*n* = 9). The CAPA type was linked to the cause of the errors, but not to a test phase [Fig. 1]. For 14 problems, no action was undertaken at all. Seven of them included problems with the tissue material [Fig. 1]. In 2016, the type of CAPA was correlated to less genotyping errors, successful participation (both *p* = 0.027), and obtaining the maximum score (*p* = 0.045) in the 2017 EQA scheme, especially for protocol revisions. Time of error registration (before or after the release of the EQA results) had no influence on the next scheme’s performance.

At laboratory level, more laboratories changed their method or protocol in 2016 compared to 2017 (Table 3). In both years, interpretation as well as reporting of the results was mainly performed by the molecular biologist and pathologist. Whereas the pathologist mainly interpreted results of commercial kits, non-commercial methods were mainly interpreted by the laboratory director.

In case a pathologist was involved in reporting, participants were more likely to obtain no genotyping errors (*p* = 0.15), a higher genotyping score (*p* = 0.034), the maximum score (*p* = 0.028), and a successful participation (*p* = 0.015) in 2017. This was not the case for the interpretation or reporting by any of the other responsible persons.

### Feedback to laboratories

Ten participants from six laboratories at different countries (Austria, Germany, Israel, Portugal, Romania, and Turkey) accepted the invitation to attend the workshop. From those six institutes who attended, four of them also completed the 2016 survey and participated again in the 2017 scheme. Attendees scored the quality and usefulness for routine implementation of the workshop at 95 and 89 on 100 points, respectively. Participants responded that the main hurdles to overcome related to biomarker testing were routine problems including time and staff constraints (8/10), organizational and institutional barriers (5/10), an increasing workload (4/10), or costs or reimbursement issues (3/10).

**Fig. 2:**
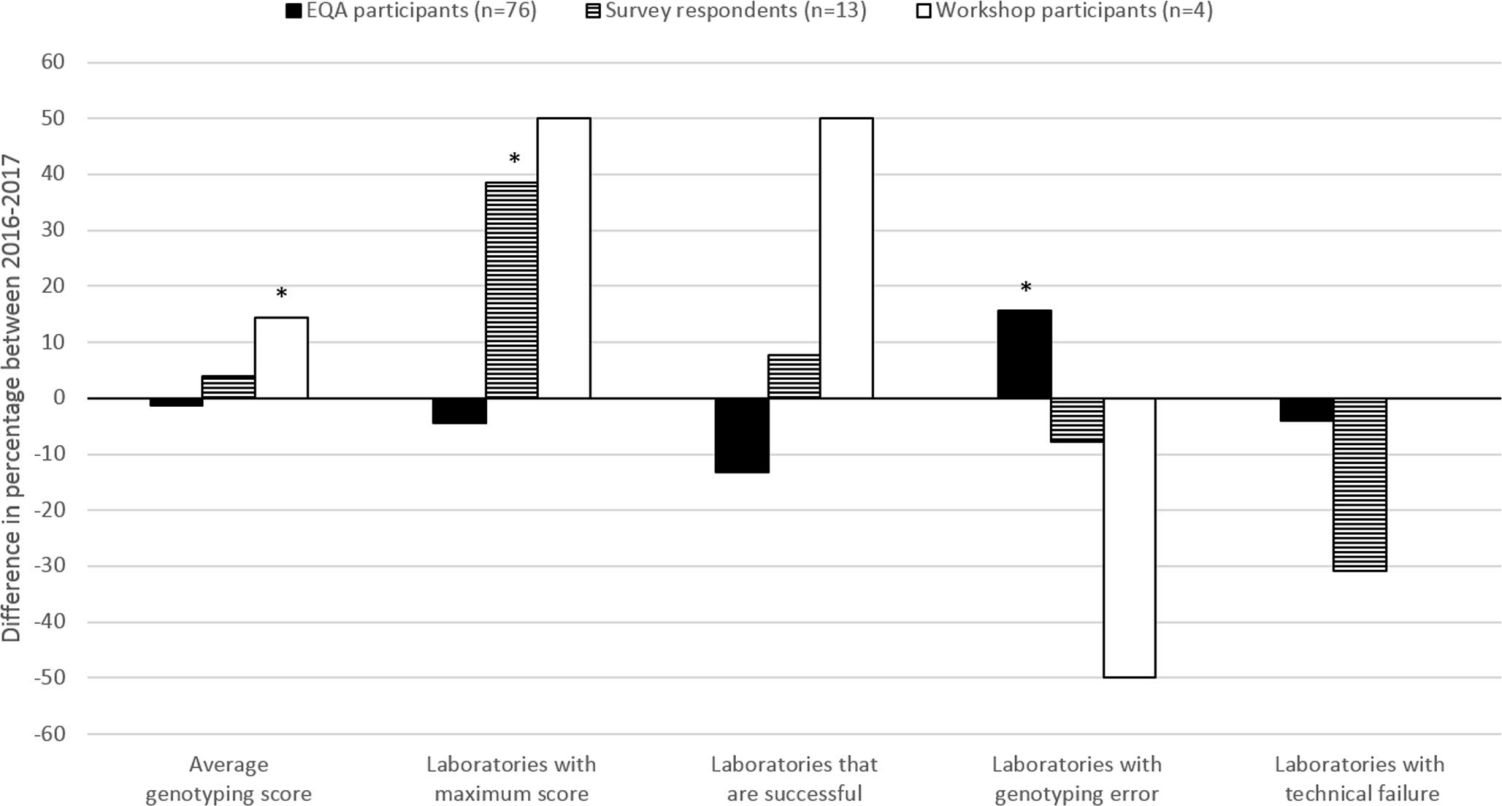
Overview of improvement between the 2016 and 2017 ESP colon EQA schemes. *p < 0.05. Only laboratories who participated in both EQA schemes were taken into account. Participants were awarded two points per case for a correct outcome, resulting in a maximum genotyping score of 20 points (23). Laboratories are considered successful if they have a genotyping score of ≥ 90%, without major genotyping errors

The improvement in 2017 was evaluated for three groups: (a) laboratories participating in both schemes who received individual feedback (*n* = 76), (b) survey respondents (*n* = 13), and (c) workshop participants (*n* = 4) (Fig. 2). The number of laboratories making a genotyping error in 2017 (*p* = 0.036) increased significantly for returning EQA participants, in contrast to survey respondents and workshop participants. For those last two groups, an increase was observed in the average genotyping score (*p* = 0.037) and the percentage of laboratories obtaining the maximum score (*p* = 0.039), respectively.

## Discussion

Accurate biomarker tests are crucial to determine appropriate treatment options for mCRC patients. To further improve the standard of biomarker testing, diagnostic laboratories are encouraged [16, 17, 24, 25] to implement measures for continual quality improvement, including CAPAs, education of laboratory personnel, and participation to EQA to compare the laboratory’s performance to peers and identify improvement priorities. This study focused on laboratories that reported an error during the annual EQA assessment. It included an in-depth analysis of EQA results to assess the influence of the exact error causes and their follow-up on performance, which have been reported in other fields besides molecular pathology [15, 18–20]. It included a survey and evaluation of customized feedback provided to the participant laboratories.

EQA participation has been shown to reflect *RAS* testing performance in routine practice [14]. This was confirmed by this study by a significant link between recurring errors and a lower average analysis score for participants who performed less in the first EQA scheme. This also suggests that EQA might exert a positive influence on laboratory performance, as previously reported for non-small cell lung cancer [21]. The results of this study demonstrated 6.1% of samples misclassified in 2017. Given the impact of the biomarker status on treatment choice it is important to continue improving biomarker testing.

Active participation to quality improvement projects aids laboratories in the critical evaluation of their results, as shown by the improved performance for workshop participants and survey respondents compared to general participants. Indeed, protocol revisions were frequently reported as CAPAs in the survey, and performing this CAPA type led to less errors and a better score in the next scheme. These revisions might be technical (e.g., a change to the analysis protocol) or general of nature (e.g., a general change to prevent errors from occurring such as building in a second check step when entering the results in to the online system). Moreover, the fact that the type of action performed (instead of type of error) influences the performance in the next scheme, suggests an active role for laboratories in quality improvement.

Survey respondents were a good representation of EQA participants, and error monitoring was not restricted to larger laboratories, laboratories in a research setting or who are accredited for molecular pathology. Although receiving accreditation was not linked to a better performance in this study, it has previously been shown to aid in the successful implementation of a new biomarker [26]. These surveys had the advantage of a standardized taxonomy, which allows to monitor error causes on a longitudinal level. The availability of multiple international laboratories’ data enables to link error causes to specific laboratory characteristics and methodologies. This can reveal systematic shortcomings and critical points in the TTP, eventually guiding molecular diagnostic laboratories.

In terms of continuous education, many laboratories did not perform additional training for results interpretation besides a person’s educational degree. Although training of the staff for a specific methodology should be well documented, and re-evaluated at frequent intervals [16], this was not reflected in the EQA performance.

Analysis of 56.2% technical failures and 40.8% of genotyping errors in both schemes combined, stresses the need of risk analysis in the TTP instead of merely the analytical phase. Consistent with previous results [15, 18], the pre- and post-analytical phases constituted a high fraction of the observed causes. In addition, as pre-cut and pre-labeled slides were provided to participants, an evaluation of the pre-analytical errors in routine practice of prior steps (deparaffinization, cutting, labeling) is advisable [27] as well as of errors at phases outside the laboratory’s responsibility. Namely, errors were reported in the pre-pre-analytical phase (from test request to sample reception at the laboratory) and the post-post-analytical phase (interpretation of the reported results by the clinician and making the appropriate therapy decision), albeit in other fields [15, 18–20].

This study demonstrates that pre-analytical errors were more likely to result in not obtaining the maximum score in the next scheme, and that a close involvement of the pathologist in results reporting and error follow-up contributes to a better scheme performance and less pre-analytical problems, especially when using a commercial kit, in line with a previous longitudinal study [13]. This stresses the importance for standardization of the neoplastic cell content determination for test outcome interpretation in mCRC [13] (Dufraing et al., submitted for publication). This is supported by the observation that only for 1 out of 12 cases for which the laboratories reported that the tumor tissue was not optimally suited for analysis in 2016, this was good practice, as it was indeed a case without neoplastic cells. Coincidently, survey respondents in 2016 were less likely to perform the analysis at the department of pathology and more frequently outsourced the selection of the neoplastic cell to another laboratory. Therefore, it might be useful to analyze a larger dataset or to evaluate non-conformities in routine practice to evaluate if these responder characteristics might have skewed the data and if non-conformities observed during routine reflect non-conformities reported during EQA, as even more challenging cases might occur in routine.

In 2017, more (analytical) methodological and personnel errors were observed, and more frequently no CAPA was implemented compared to the pre-analytical issues reported in 2016. This is surprising, as this is a requirement of the ISO 15189 and similar quality framework. Also, CAPAs were more likely to be monitored by the laboratory director at the expense of the laboratory technicians and were performed more often before the official release of the EQA results. This might suggest that these analytical problems are considered to be more severe by participants as compared to pre- and post-analytical problems in 2016, and direct follow-up may be more difficult. Indeed, reported causes included (a) unknown factors for which the manufacturer needed to be contacted or (b) a variant that was not included in the method for analysis, not linked to a specific methodology. Surprisingly, in spite of the large number of NGS users, none of the laboratories included a bio-informatician to interpret the results [28]. However, it must be noted that not all survey respondents in 2016 (*n* = 24) registered again in the next EQA scheme of 2017, as yearly participation is not required to demonstrate high quality performance. Therefore, we contacted those participants (*n* = 8) to ask for the reasons of refraining from participation. Two laboratories mentioned they only participate once every 2 years. One laboratory merged with another institute and therefore stopped *RAS* analyses, while another laboratory experienced bureaucratic issues with the payment of the registration fee. One participant did not agree with their awarded analysis score in 2016. The other three laboratories did not respond.

To interpret the error rates in the EQA schemes there are four points that need to be taken into account: (a) More samples were included containing a *KRAS* variant. However, no differences were observed when re-calculating the error rates based on the number of included samples per gene. (b) The ten distributed samples each had a different origin. Error rates were highest for a case containing the c.436G > A p.(Ala146Thr) variant (15.4%, *n* = 123) in 2016, and for the c.176C > A p.(Ala59Glu) variant (24.8%, *n* = 105) in 2017. The reason is that laboratories may be using an analysis method which may not include all necessary codons, consistent with previous EQA schemes [10]. (c) Many laboratories incorrectly analyzed the sample without any neoplastic cells, for which numbers and consequences have been previously described [13]. (d) Pre-defined scoring criteria differed as laboratories with an error in the online datasheet but correct written report received full points in 2017 and no points in 2016. However, this had no influence on the scores in this study.

As a conclusion, quality improvement projects such as the study described here are important to further improve the current high standards of biomarker testing in Europe. To avoid any issues with testing, laboratories need to work according to pre-defined procedures and document any changes. Laboratories need to be aware that reporting and monitoring of errors is required for quality improvement. To assure quality of biomarker analysis, it is thus clear that a holistic approach [29] is needed at all phases in combination with quality improvement projects within the laboratory and organized by EQA providers.

## Electronic supplementary material

ESM 1 (PDF 318 kb)

ESM 2 (PDF 244 kb)

## Data Availability

The datasets generated during and/or analyzed during the current study are available from the corresponding author on reasonable request.

## Abbreviations

*BRAF*: B-Raf proto-oncogene
*EGFR*: Epidermal growth factor receptor
ESP: European Society of Pathology
FE: Fisher’s exact test
*KRAS*: Kirsten rat sarcoma viral oncogene homolog
mCRC: Metastatic colorectal carcinoma
MWU: Mann-Whitney *U* test
*NGS*: Next-generation sequencing
*NRAS*: neuroblastoma rat sarcoma
WT: Wild-type
*Χ*^2^: Chi-squared test
