# Supplementary material for: European follow-up of incorrect biomarker results for colorectal cancer demonstrates the importance of quality improvement projects
Source: Virchows Arch. 2019 Feb 5;475(1):25–37. doi: 10.1007/s00428-019-02525-9 (PMC6611891; doi:10.1007/s00428-019-02525-9)
Supplement: Supplementary file 1 — (PDF 318 kb) [file 428_2019_2525_MOESM1_ESM.pdf]

Title	European follow-up of incorrect biomarker results for colorectal cancer demonstrates the importance of quality improvement projects.
Journal	Virchows Archiv

Authors	Keppens Cleo, Dufraing Kelly, van Krieken J Han, Siebers G Albert, Kafatos George, Lowe Kimberly, Demonty Gaston, Dequeker MC Elisabeth
Correspondence	Prof. Dr. Elisabeth Dequeker, Department of Public Health and Primary Care, Biomedical Quality Assurance Research Unit, University of Leuven, Kapucijnenvoer 35d, Box 7001, Leuven 3000, Belgium. Tel: +3216 345881, Fax: +3216 347949. E-mail: els.dequeker@kuleuven.be

File

Supplemental Table 1: Overview of the samples provided during the 2016 and 2017 ESP colon EQA schemes.

Participating laboratories received three slides of ten formalin-fixed paraffin embedded cases for analysis by their routine procedures. In 2016, the preparation and validation of the samples was done by 8 scheme organizers (Each organizer prepared, validated, and sent samples for approximately 16 participants). In 2017, this was performed by one central reference laboratory, and confirmed by a second independent laboratory.

Reference sequences used at the time of analysis: *KRAS*: NM_033360.3; NM_004985.4. *NRAS*: LRG_92 (NM_002524.3), *BRAF*: LRG_299 (NM_004333.4).

Abbreviations: *BRAF*: B-Raf proto-oncogene, *KRAS*: Kirsten rat sarcoma viral oncogene homolog, *NRAS*: neuroblastoma rat sarcoma, WT: Wild-type.

Scheme year	2016				2017		
Sample number	Subscheme	*KRAS*	*NRAS*	*BRAF*	*KRAS*	*NRAS*	*BRAF*
1	A, B, D, E, F	WT	c.181C>A p.(Gln61Lys)	WT	Sample without neoplastic cells	Sample without neoplastic cells	Sample without neoplastic cells
	G, H		c.182A>T p.(Gln61Leu)				
	C		c.182A>G p.(Gln61Arg)				
2	All	WT	WT	WT	c.176C>A p.(Ala59Glu)	WT	WT
3	All	Sample without neoplastic cells	Sample without neoplastic cells	Sample without neoplastic cells	WT	WT	WT
	D	WT	WT	WT			
4	All	c.35G>T p.(Gly12Val)	WT	WT	WT	WT	c.1799T>A p.(Val600Glu)
5	All	c.38G>A p.(Gly13Asp)	WT	WT	WT	c.182A>T p.(Gln61Leu)	WT
6	All	c.436G>A p.(Ala146Thr)	WT	WT	WT	WT	WT
7	All	WT	WT	WT	c.35G>A p.(Gly12Asp)	WT	WT
8	All	c.35G>A p.(Gly12Asp)	WT	WT	WT	WT	WT
	C	c.35G>A p.(Gly12Asp) and c.351A>T p.(Lys117Asn)					
9	All	WT	WT	c.1799T>A p.(Val600Glu)	c.35G>C p.(Gly12Ala)	WT	WT
10	All	WT	WT	WT	c.38G>A p.(Gly13Asp)	WT	WT
