# Supplementary material for: European follow-up of incorrect biomarker results for colorectal cancer demonstrates the importance of quality improvement projects
Source: Virchows Arch. 2019 Feb 5;475(1):25–37. doi: 10.1007/s00428-019-02525-9 (PMC6611891; doi:10.1007/s00428-019-02525-9)
Supplement: Supplementary file 2 — (PDF 244 kb) [file 428_2019_2525_MOESM2_ESM.pdf]

Title	European follow-up of incorrect biomarker results for colorectal cancer demonstrates the importance of quality improvement projects.
Journal	Virchows Archiv
Authors	Keppens Cleo, Dufraing Kelly, van Krieken J Han, Siebers G Albert, Kafatos George, Lowe Kimberly, Demonty Gaston, Dequeker MC Elisabeth
Correspondence	Prof. Dr. Elisabeth Dequeker, Department of Public Health and Primary Care, Biomedical Quality Assurance Research Unit, University of Leuven, Kapucijnenvoer 35d, Box 7001, Leuven 3000, Belgium. Tel: +3216 345881, Fax: +3216 347949. E-mail: els.dequeker@kuleuven.be
File	**Supplemental Table 2: Overview of definitions for the error categories available in the survey and examples reported by respondents.**

Category	Included criteria	Real life examples from survey respondents
Pre-analytical phase	Sample reception, sample identification, sample preparation: deparaffinization, H&E staining, micro/macrodissection, selection of neoplastic cell region by pathologist, scraping of the tissue	
Analytical Phase	DNA extraction, library preparation, mutation analysis according to pre-determined protocol	
Post-analytical phase	Readout of the analytical results, interpretation of the samples according to predetermined criteria, clinical interpretation, communication of results , entering of the results in the laboratory information system/EQA datasheet	
Clerical error	Sample processed correctly, but results were incorrectly entered into the EQA datasheet or the laboratory's own information system/miscommunication between departments.	▪ Mistake by manually entering the result into the online datasheet<br>▪ Result was negative but was incorrectly reported as positive
Interpretation error	Sample processed correctly, but incorrect conclusions are made from the output of the test.	▪ Mutated peak was closer to the positive control in a WT case<br>▪ Artefact substitution (G>A) in a hotspot was considered as a true mutation<br>▪ Incorrect filter applied to the variant list
Methodological problem	Problems that occur because the method is not optimally suited for all situations presented at the laboratory.	▪ Variant of the gene was not detected by the kit for unknown reasons<br>▪ Gene variant was not included in the kit or frequency was below limit of detection<br>▪ Occasional problem with reporting of low frequent variants by the method
Personnel error	Error that occurred due to a mistake of the personnel (besides clerical errors).	▪ Specimen mix-up<br>▪ Pipetting error during PCR<br>▪ Probably contamination by another sample<br>▪ Rare variant, clinical significance overseen in output due to time pressure
Problem with tissue material	Problems that are caused because the EQA material differs from material used for validation of the method.	▪ Insufficient amount of library and subsequently, insufficient coverage<br>▪ Fragmented DNA<br>▪ No efficient amplification of the PCR product
Reagent problem	Unexpected problems that occur with reagents for the analysis.	▪ Suspected reagent problem in commercial kit (not further specified)
Technical problem	A technical problem occurred with the available hardware/software present in the laboratory.	▪ Heat-plate for annealing was not calibrated well<br>▪ Analysis error in the used software plug in<br>▪ Sequencer unable to analyze the sequence because the DNA template was not good enough
